# Utilizing Three-Dimensional Culture Methods to Improve High-Throughput Drug Screening in Anaplastic Thyroid Carcinoma

**DOI:** 10.3390/cancers14081855

**Published:** 2022-04-07

**Authors:** Kensey Bergdorf, Joshua A. Bauer, David Westover, Courtney Phifer, Barbara Murphy, Darren R. Tyson, Ethan Lee, Vivian L. Weiss

**Affiliations:** 1 Department of Pharmacology, Vanderbilt University, Nashville, TN 37232, USA; kensey.n.bergdorf@vanderbilt.edu (K.B.); ethan.lee@vanderbilt.edu (E.L.); 2 Department of Biochemistry, Vanderbilt University, Nashville, TN 37232, USA; joshua.a.bauer@vanderbilt.edu (J.A.B.); darren.tyson@vanderbilt.edu (D.R.T.); 3 Vanderbilt Institute of Chemical Biology-High-Throughput Screening Facility, Vanderbilt University, Nashville, TN 37232, USA; david.westover@merck.com; 4 Vanderbilt Ingram Cancer Center, Nashville, TN 37232, USA; barbara.murphy@vumc.org; 5 Department of Pathology, Microbiology, and Immunology, Vanderbilt University Medical Center, Nashville, TN 37232, USA; courtney.j.phifer@vumc.org; 6 Department of Cell and Developmental Biology, Vanderbilt University, Nashville, TN 37232, USA

**Keywords:** anaplastic thyroid carcinoma, high-throughput screening, spheroids, therapeutics

## Abstract

**Simple Summary:**

There are currently few treatment options for individuals diagnosed with anaplastic thyroid carcinoma (ATC). Using four distinct ATC cell lines, we screened over 1500 anti-cancer agents and FDA-approved drugs. The initial screen and secondary confirmation testing identified 40 agents of interest for further evaluation. Validation was performed using three-dimensional anaplastic thyroid carcinoma cell cultures (spheroids) in order to more closely recapitulate in vivo drug response. Our approach has enabled identification of three exceptionally potent compounds, bortezomib, cabazitaxel, and YM155, and enhanced in vivo translatability to inform future clinical trials.

**Abstract:**

Anaplastic thyroid carcinoma (ATC) is the most aggressive endocrine neoplasm, with a median survival of just four to six months post-diagnosis. Even with surgical and chemotherapeutic interventions, the five-year survival rate is less than 5%. Although combination dabrafenib/trametinib therapy was recently approved for treatment of the ~25% of ATCs harboring *BRAFV600E* mutations, there are no approved, effective treatments for *BRAF*-wildtype disease. Herein, we perform a screen of 1525 drugs and evaluate therapeutic candidates using monolayer cell lines and four corresponding spheroid models of anaplastic thyroid carcinoma. We utilize three-dimensional culture methods, as they have been shown to more accurately recapitulate tumor responses in vivo. These three-dimensional cultures include four distinct ATC spheroid lines representing unique morphology and mutational drivers to provide drug prioritization that will be more readily translatable to the clinic. Using this screen, we identify three exceptionally potent compounds (bortezomib, cabazitaxel, and YM155) that have established safety profiles and could potentially be moved into clinical trial for the treatment of anaplastic thyroid carcinoma, a disease with few treatment options.

## 1. Introduction

Anaplastic thyroid carcinomas (ATCs) are exceptionally aggressive tumors, with a median survival time of 4–6 months post-diagnosis, a 35% six-month survival rate, and a dismal five-year survival rate of less than 5% [1,2]. These de-differentiated tumors grow rapidly and have a disease-specific mortality nearing 100% [3]. As the genetic and molecular drivers of transformation to ATC are not well-understood, early detection and treatment options are limited. It is this disparity, combined with their aggressive nature, that leads ATCs to account for over half of all thyroid-cancer-related deaths despite comprising fewer than 2% of all thyroid neoplasms [4].

Approximately 25% of ATCs harbor a *BRAFV600E* mutation, which leads to constitutive activation of MAPK signaling. Fortunately, mutant *BRAF* can be targeted using dabrafenib, and paradoxical MEK/ERK activation can be avoided with the addition of trametinib [5,6]. This combination therapy was FDA-approved for the treatment of *BRAFV600E* mutant ATC in 2018. Median overall survival in patients treated with dabrafenib-trametinib therapy was extended to 14.5 months, though only 8% of patients exhibited a complete response to the therapy [6]. There are currently no effective, targeted therapies available for *BRAF*-wildtype ATC.

We have recently developed methods for culturing thyroid cancer spheroids and adapting these cultures to formats amenable to high-throughput drug screening [7]. Spheroids allow for cell–cell interactions and nutrient gradients that are not possible in a traditional monolayer culture, making them an ideal model for drug discovery and development [8,9]. Additionally, drug responses in three-dimensional cultures have previously been shown to deviate from those observed in corresponding monolayer cultures and to more accurately recapitulate patient tumor responses [10,11].

In this study, we sought to combine our ATC spheroid cultures with high-throughput drug screening to identify potential therapeutics for a disease with few treatment options. Through this methodology, we were able to identify three lead candidates for follow-up studies in both *BRAF*-mutant and -wildtype ATC. Notably, this study provides the basis for many routes of investigation into the molecular drivers and therapeutic targets of ATC to inform future clinical trials.

## 2. Materials and Methods

Cell Culture. ATC cell lines (THJ-11T, THJ-16T, THJ-21T, and THJ-29T) were obtained from Dr. John Copland (Mayo Clinic, Jacksonville, FL, USA). Cell lines are maintained at 37 °C, 5% CO_2_ in RPMI (VWR, Radnor, PA, USA) supplemented with 10% fetal bovine serum (ThermoFisher Scientific, Waltham, MA, USA), 1% penicillin-streptomycin (Sigma, St. Louis, MO, USA), 1X MEM non-essential amino acids (VWR), and 1 mM sodium pyruvate (hereafter referred to as “complete RPMI”). All cell lines are used experimentally at less than 20 passages and authenticated via STR analysis.

Drug libraries. The NCI Approved Oncology Drugs Set VII (National Cancer Institute, Division of Cancer Treatment and Diagnosis, Developmental Therapeutics Program) and the Selleck Chemicals FDA-approved and Anti-cancer Compound libraries (Selleck Chemicals, Houston, TX, USA) are maintained and distributed by the Vanderbilt High-Throughput Screening (VHTS) facility within the Vanderbilt Institute of Chemical Biology. In total, 1525 compounds were screened.

Primary high-content imaging screen in ATC cell lines. High-throughput screens were performed in collaboration with the VHTS facility. Cells were seeded in 384-well cell culture plates (Greiner Bio-One #781091, Greiner Bio-One, Kremsmünster, Austria) at a density of 600 cells/well (THJ-11T; THJ-16T; THJ-29T) or 900 cells/well (THJ-21T) using a ThermoScientific Multi-Drop Combi dispenser and incubated at 37 °C with 5% CO_2_. After 24 h, drugs and small molecules were transferred from library stock plates at 10 mM DMSO solutions using an ECHO acoustic liquid transfer system (LabCyte/Beckman) to a 384-well drug plate. Drug plates were diluted with complete RPMI and robotically added to the cell plates at five final concentrations (5.14 µM, 1.09 µM, 156 nM, 30.8 nM, 6.2 nM) in 0.2% DMSO (*v*/*v*). These ~5-fold dilutions allowed for a representative curve to be generated for drugs with potency ranging from low micromolar through nanomolar concentrations. Cells were incubated with drugs for an additional 72 h, after which they were stained with Hoechst 33,342 (Invitrogen, Waltham, MA, USA) and propidium iodide (MilliporeSigma, Burlington, MA, USA) to final concentrations of 2 µg/mL and 0.2 µg/mL, respectively, and imaged on an ImageXpress Micro XLS using DAPI and Texas red filters (Molecular Devices, LLC, San Jose, CA, USA).

**Figure 1:**
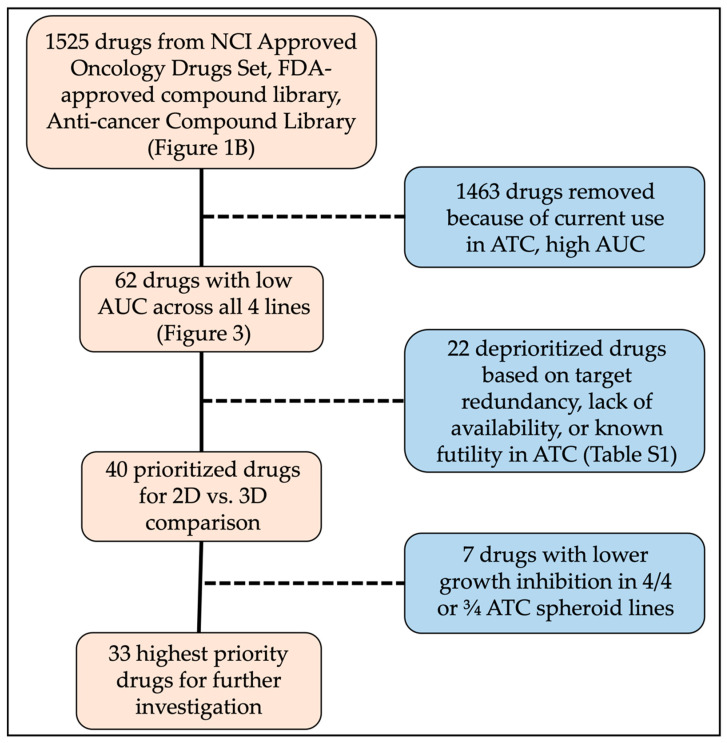
Schematic of drug numbers throughout studies.

**Figure 2:**
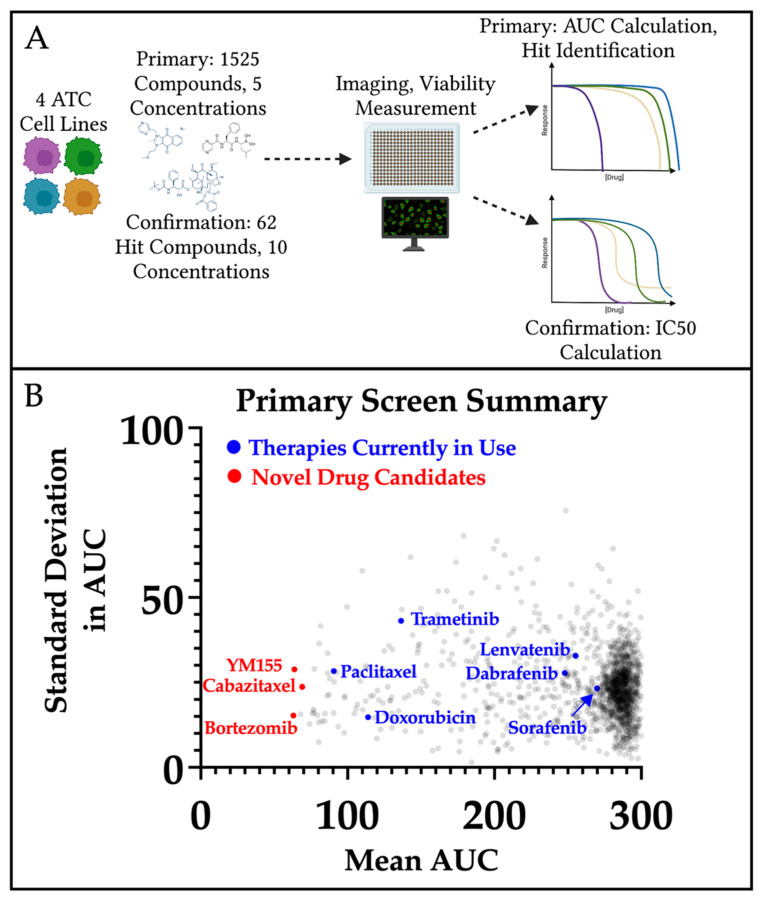
Workflow and results of primary compound screen. (**A**) Schematic of plating, treating, and analysis for the primary compound screen. The four ATC cell lines (THJ-11T, THJ-16T, THJ-21T, and THJ-29T) were plated in 384-well tissue culture plates. After a 24 h incubation, 1525 compounds were added to plates at five concentrations (5.14 µM, 1.09 µM, 156 nM, 30.8 nM, 6.2 nM). Plates were incubated for 3 days prior to staining for the nucleus and dead/dying cells and imaging. (**B**) Summary results for primary compound screen. Curves were generated for each compound (for each cell line), and average area under the curve (AUC) and standard deviation across the four lines were calculated for each compound. Each point on the plot represents the results of one drug across all four ATC cell lines. Therapies that are currently in use or clinical trial are denoted by blue points, and compounds that showed low AUC and low standard deviation are denoted by red points.

**Figure 3:**
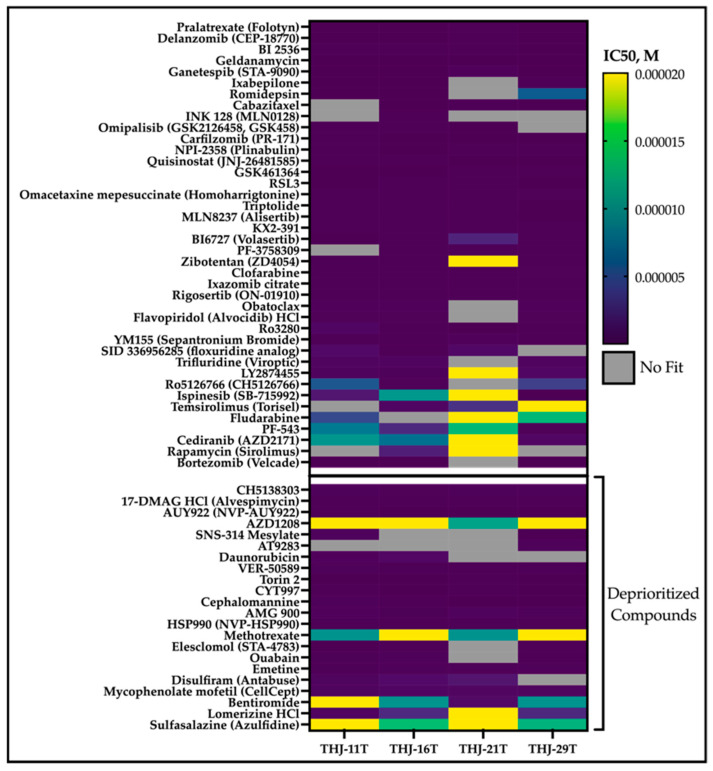
Summary of confirmation screen. Ten concentrations of each compound were plated with each of the four ATC cell lines and incubated for 3 days prior to imaging to detect nuclei and dead cells. High potency (low IC50) drugs are denoted in purple, and lower potency drugs are denoted in the green/yellow cells. Cells are shaded in grey if no curves could be generated to fit the collected data. Drugs that exhibit target redundancy, lack of current clinical availability, and/or known futility in ATC are graphed separately as deprioritized compounds.

Viable cells were identified as those that were Hoechst positive and propidium iodide negative and compared across cell lines for each drug using the area under the curve for the 5 concentrations. In total, 62 drugs were identified via AUC (Figure 2) and further confirmed by concentration-response plots generated using ten concentrations in technical triplicate ranging from 20 µM to 20 nM (Figure 3). Overall, 22 drugs did not progress to further studies due to: (1) target redundancy; (2) lack of current clinical availability; or (3) established clinical futility in ATC (Figure 1). A full list of eliminated compounds is provided in Supplementary Table S1.

Lentiviral transduction. A lentivirus containing constitutive nuclear mKate under the human PGK promoter with blasticidin selection (TRC2-pLKO-PGK-nlsmKate2/Bsd) was generously provided by Dr. Huan Qiao (Vanderbilt University, Nashville, TN, USA). All cell lines were transduced at a multiplicity of infection of 10:1 for 24 h prior to media replacement. Blasticidin selection concentration was determined prior to transduction and defined as the concentration needed to kill non-transduced cells in 5 days. Final concentrations used were between 5 and 10 µg/mL. Selection media (complete RPMI + blasticidin) were changed every 48 h for the first two weeks post-transduction, then passaged regularly and maintained in the selection media as needed.

Comparison of time-resolved drug response in 2D vs. 3D growth conditions. Transduced cells were seeded for monolayer culture in 384-well cell culture plates (Greiner Bio-One #781091) at a density of 300 cells/well in complete RPMI. All cell lines were seeded for spheroid culture in 384-well cell-repellent culture plates (Greiner Bio-One #781976) at a density of 600 cells/well (THJ-11T; THJ-16T; THJ-29T) or 900 cells/well (THJ-21T) in complete RPMI supplemented with 2% Matrigel (Corning, Corning, NY, USA) using a peristaltic EL406 dispenser (BioTek, Santa Clara, CA, USA) within a biosafety cabinet. Plates were immediately centrifuged for 5 min at 200 g to allow cells and matrix to collect on the flat bottom plates. Following a 24-h incubation at 37 °C, 5% CO_2_, cell plates were placed into an automated incubator (Cytomat, Thermo) and each plate was shuttled to a Molecular Devices’ ImageXpress MicroXL imaging system via a robotic plate handler (F3 arm, Thermo) and using the Momentum scheduler. Following initial cell density baseline imaging, drug plates diluted in media (as above) plus 5 nM SYTOX Green (to detect dead cells—Invitrogen) were added to cell plates using a liquid handler and returned to the Cytomat. Cell plates were scheduled in a continuous loop to be imaged consecutively for 5 days.

Analysis of 2D vs. 3D drug response data. The number of viable cells at each time point was determined by counting segmented nuclei from the red fluorescence channel that did not have fluorescent signal overlap in the green (dead cell) channel using custom Python scripts as previously described [12]. We calculated the highest dead cell fraction observed at any point over 72 h at a given drug concentration, and that value was subtracted from 1 to generate a fraction of viable cells. These data were uploaded into a local instance of Thunor-web software that was specifically designed to automatically fit the data with 4-parameter log-logistic models [13]. The activity area (observed) is effectively the integrated area above the observed response values and calculated as described (https://docs.thunor.net/dose-response-parameters, accessed on 13 February 2022) [13]. Observed activity area (AAobs) is used in the analyses of these data rather than AUC, as AAobs is positively associated with drug efficacy, where AUC is an inverse association. AAobs is also independent of any fitting function, relying only on the data collected. This same approach was applied to the spheroid cultures after first obtaining a maximum projection image from the complete z-stack obtained for each sample and time point.

**Figure 4:**
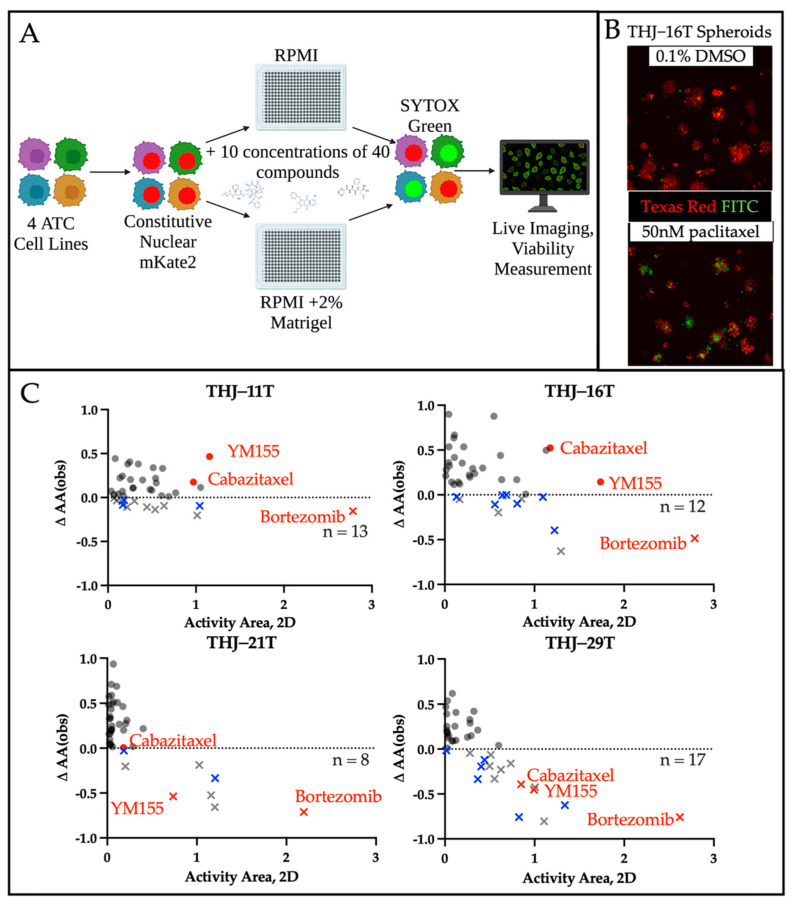
Inclusion of spheroids in drug screening workflows identifies drugs with culture-format dependent effects. (**A**) Schematic of workflow for the monolayer vs. spheroid screen. (**B**) ATC cell lines were infected with lentiviruses to express nuclear mKate2 (Texas Red channel) prior to plating in 384-well plates and treating with 40 priority compounds over a range of 10 concentrations. SYTOX Green was included to mark dead or dying cells (FITC channel, green). With DMSO treatment, baseline cell death can be observed at the margins of each spheroid. When treated with 50 nM paclitaxel, the ratio of dead to live cells visibly increases. (**C**) Comparison of 2D and 3D culture response. Positive values (circles) represent drugs with retained/enhanced inhibition in spheroids, whereas negative values (×) indicate drugs that demonstrate greater inhibitory activity in monolayer cultures. Counts represent the number of drugs (of the 40 drugs tested) that exhibit decreased inhibition in spheroids, and blue (×)s correspond to drugs with decreased responses in ≥3 spheroid lines.

To define differential drug response between monolayer and spheroids, AAobs values were plotted on axes of 2D AAobs (x) vs. 3D AAobs (y) (Supplemental Figure S3). If a given compound produced identical results in monolayer and spheroid cultures, we expect that point to fall at x = y. A reference line was drawn representing this relationship and residuals from this reference were plotted for each cell line (Figure 4C—deltaAA). A positive value indicates higher activity in spheroid culture, whereas a negative value indicates increased activity in monolayer cultures. At this point, drugs were eliminated if they exhibited decreased inhibition in at least three of the four ATC spheroid cultures (Supplemental Table S2).

## 3. Results

### 3.1. Primary and Confirmation Screens Identify 40 High-Priority Compounds

#### 3.1.1. Primary Compound Screen of 1525 Drugs Identifies 62 Hits

For our studies, we utilized four anaplastic thyroid carcinoma cell lines—THJ-11T; THJ-16T; THJ-21T; and THJ-29T. These cell lines were chosen due to distinct mutational and morphological profiles, summarized in Table 1, that are representative of disease diversity observed in vivo [14,15]. In using these distinct lines, we sought to identify compounds that were effective in inhibiting (1) ATC lines harboring certain mutations (*BRAFV600E*, *KRAS*, *PI3KCA*); (2) ATC lines harboring wildtype *BRAF*; and (3) all lines regardless of mutational status.

We first executed a screen of 1525 compounds from three established libraries: NCI Approved Oncology Drug Set VI, FDA-Approved Drug Collection, and the Anti-Cancer Compound Library. Five concentrations of each compound were tested in the four cell lines in monolayer culture (Figure 2A), and results were reported as area under the curve (AUC). As expected, many compounds did not significantly inhibit growth in any of the four ATC lines and clustered at the far right of the plot (Figure 2B). However, we were able to confirm the inhibitory effects of currently used therapeutics such as paclitaxel, doxorubicin, and trametinib (blue—Figure 2B). Notably, tyrosine kinase inhibitors lenvatinib and sorafenib were not broadly effective across the four lines (Figure 2B). For prioritization, drug hits were identified as those with the lowest AUC and lowest standard deviation in AUC. In total, we identified 62 compounds of interest to perform a follow-up confirmation screen. Drug classes represented in the 62 prioritized compounds included inhibitors of HSP90, HDAC, and proteasomes, all of which have been investigated for the treatment of ATC in recent years [2]. Our results confirm the importance of these pathways in ATC and their identification provided increased confidence in the results of our broad screen.

#### 3.1.2. Confirmation Screen Allows for IC50 Calculation and Prioritization

To further prioritize compounds for more in-depth follow-up, we generated dose response curves for 62 compounds in monolayer culture of the four ATC cell lines (Figure 3). Following confirmation of efficacy, we evaluated compounds based on potency, clinical trial success, previous relevance in ATC, and target redundancy. This resulted in removal of 22 compounds, including emetine (an emetic), disulfiram (Antabuse, a treatment for alcohol dependence), and four of seven HSP90 inhibitors. A full list of eliminated compounds can be found in Supplemental Table S1. Finally, we were left with 40 priority drugs to compare in monolayer and spheroid cultures.

### 3.2. Three-Dimensional Culture Identifies Compounds with Differential Efficacy

#### 3.2.1. Value of Spheroids in Drug Screening

Spheroids are three-dimensional cell structures composed of a single cell source. We previously reported the development and characterization of multiple thyroid cancer spheroid lines, as well as the methods for adapting them to high-throughput formats [7]. Briefly, our spheroids are derived from monolayer cultures and grow successfully in low-attachment or cell-repellent plates in as little as 2% Matrigel. Structures can vary in size and morphology, with THJ-16T spheroids being relatively large (200–400 µm) whereas compact and THJ-21T spheroids are smaller (50–100 µm) and less cohesive [7] (Supplemental Figure S1). Although these differences cause concern for issues of drug penetrance, we do not observe any correlation between spheroid size and consistently diminished potency or efficacy in three-dimensional cultures. As spheroids more accurately recreate nutrient and oxygen gradients that occur in vivo, they are believed to better represent patient responses to therapeutics. Due to the cost and time associated with spheroid screens, we understand that they are not viable options for many initial screens. However, in adapting these cultures to a high-throughput format to decrease time required and using a minimal Matrigel content to reduce costs, we aim to enhance the feasibility and translatability of these data.

#### 3.2.2. Comparing Monolayer and Spheroid Drug Responses to Inform Future Studies

We tested our 40 priority drugs, which were identified following deprioritization of 22 drugs from the confirmation screen, in all four ATC cell lines in both monolayer and spheroid cultures to identify those with altered efficacy in three-dimensions (Figure 4A). mKate2-labeled cancer cells (Figure 4B, Supplemental Figure S1) were exposed to SYTOX Green to identify dead/dying cells. Relative viability at each drug concentration was plotted for each drug, cell line, and culture condition, allowing for identification of those with diminished response in spheroids (Supplemental Figure S2). The activity area (area over the dose–proliferation response curve) for each drug in each cell line indicated seven compounds with decreased inhibitory activity in spheroid culture when compared with the corresponding monolayer culture in at least three ATC cell lines (blue—Figure 4C, Supplemental Table S2). The remaining 33 compounds showed relatively good potency and 3D inhibitory activity. Of these, we identify three drugs that have high potential for translation to clinical trial: bortezomib, a proteasomal inhibitor; cabazitaxel, a derivative of docetaxel; and YM155, a survivin inhibitor (highlighted in red, Figure 2B and Figure 4C, Supplemental Figure S4).

Although all 33 drugs with good 3D efficacy have potential therapeutic utility, we identify three with exceptional potency across all four ATC lines. Bortezomib had the highest potential for therapeutic efficacy overall. Of all the drug classes included in this study, proteasome inhibitors (bortezomib, ixazomib, delazomib, carfilzomib) demonstrated some of the highest potency across all four ATC lines. Although bortezomib demonstrated a decreased response in spheroids, it still showed the highest potency of all drugs evaluated in both monolayer and spheroid cultures (Supplemental Figure S3). Beyond the proteasome inhibitors, two additional potent drugs showed 3D efficacy in at least two of the four ATC lines, cabazitaxel and YM155. Of note, all three drugs were among the 10 top compounds identified in our initial screen (Figure 2B) based on the sum of observed activity areas across each of the four ATC cell lines.

## 4. Discussion

Three-dimensional cultures have been shown to produce differential drug responses compared with the corresponding monolayer culture. They also more accurately recreate the nutrient and oxygen gradients seen in tumors in vivo. As such, a major goal of this study was to identify novel therapeutics for anaplastic thyroid carcinoma with retained or enhanced efficacy in spheroids, rather than those that may have been over-emphasized as a result of monolayer-only screens. We utilized four unique spheroid cultures that represent a range of mutations commonly observed in ATCs to narrow the scope and list of potential therapeutic candidates from a screen of 1525 drugs. As a result, we identified multiple broadly effective therapeutic candidates that retain inhibitory activity in 3D cultures.

The vast majority of preclinical drug screening occurs in monolayer culture, as it has been the standard for decades and is highly accessible and amenable to many formats. However, with the current anti-cancer drug attrition rate of up to 95%, there is a clear need for improvement of our preclinical models of disease and their utilization in the drug development pipeline [16,17,18]. Many improvements have been made in animal models of disease, from primary xenograft models, spontaneous tumor models, and even humanized mice. However, these models are very expensive and require significant time and resources to maintain for drug studies. Our spheroid screening methodology represents a cost-effective intermediate between monolayer screening and animal tumor models to enable enhanced translatability.

There are currently no approved targeted treatments that are effective in *BRAF*-wildtype ATC. Even with the most aggressive surgical, radiation, and chemotherapeutic interventions, most patients die within 5 years [19,20]. Herein, we describe multiple compounds that demonstrate efficacy in three *BRAF*-wildtype lines with various driver mutations and one *BRAF*-mutant ATC line. Based on the initial screening data and support from follow-up three-dimensional studies, we have chosen to highlight three compounds with exceptional promise for future development: bortezomib, cabazitaxel, and YM155. All three were identified in the top 10 compounds based on total observed activity area. Bortezomib was chosen based upon having the highest consistent overall efficacy and potency; cabazitaxel has potential to improve on a current treatment for ATC (paclitaxel); and YM155 has been previously shown to inhibit ATC xenograft tumors in vivo.

Bortezomib (PS-341) is an inhibitor of the 20S core catalytic component of the 26S proteasome, which leads to accumulation of polyubiquitinated proteins and eventual apoptosis of rapidly dividing cells [21,22]. Bortezomib is currently FDA-approved for use in patients with treatment-resistant multiple myeloma and has demonstrated a manageable side-effect profile when given in an outpatient setting [23,24,25]. Bortezomib has been previously evaluated in anaplastic thyroid carcinoma, causing G(2)-M cell cycle arrest, sensitization to *BRAFV600E*-inhibitor vemurafenib, and altered tumor metabolism in vivo [26,27,28].

Cabazitaxel is a microtubule inhibitor similar to paclitaxel, which is currently used in palliative care for ATC patients [29,30]. Cabazitaxel is an exciting new therapeutic, as it exhibits a lower affinity to p-glycoprotein (Pgp, MDR1), which is known to mediate resistance to other taxanes, including paclitaxel [31]. As increased expression of MDR1 has been described in ATCs, cabazitaxel is likely to be more effective than paclitaxel in the treatment of ATCs [32]. This is supported by the results of our primary drug screen, where cabazitaxel demonstrated a consistently lower AUC across all four ATC cell lines than paclitaxel (Figure 2B). Cabazitaxel has demonstrated efficacy in metastatic taxane-resistant prostate and breast cancers but has not yet been evaluated in the treatment of ATC to our knowledge [33,34].

The final compound, YM155 (sepantronium bromide), is a potent inhibitor of the survivin promoter, with an IC50 of less than 1 nM [35]. Survivin is a member of the inhibitor of apoptosis family of proteins and has been shown to be significantly upregulated in all thyroid cancers [36]. Evaluation of ATCs has indicated that survivin is not only generally dysregulated, but has distinct nuclear localization not seen in more indolent tumor types [37]. Finally, YM155 has been characterized by others as a potential therapeutic for ATC, initiating cell cycle arrest in vitro and inhibiting the growth of ATC lung metastases in a murine xenograft model [38,39].

## 5. Conclusions

In demonstrating the importance of including three-dimensional cultures in a high-throughput compound screening workflow, we have identified 33 promising therapeutic candidates for anaplastic thyroid carcinoma. Although each of these will require further investigation prior to clinical studies, many have already successfully cleared safety testing and are in clinical trials for other tumor types. Additionally, our three lead candidates identified based on their overall potency and retained efficacy in 3D culture show promising therapeutic efficacy for ATC based on recent publications showing efficacy in mouse models, current use of the parent drug, and known relevant molecular alterations. The independent identification of compounds that have shown efficacy in separate studies using more costly mouse models of ATC validates the utility of our screening methodology. The benefits of this study are two-fold: (1) we have generated a substantial list of potentially useful drugs that already have FDA-approval and/or a history of use that can be further explored in follow up studies of ATC in vivo; and (2) we have identified three lead drug candidates that have evidence of in vivo efficacy and may be rapidly moved into clinical trials.

## Figures and Tables

**Table 1:** Mutations in and morphology of anaplastic thyroid carcinoma cell lines.

Cell Line	Notable Mutations	Morphology
THJ-11T	*KRAS c.G35T*, *TP53 c.G733A*, *TERT c.C228T* and *c.T349C*	Squamoid; compact spheroids
THJ-16T	*PI3KCA c.G1633A*, *TP53 c.G818A*, *TERT c.C228T*, *MKRN1-BRAF* fusion	Spindle; compact spheroids
THJ-21T	*BRAF c.T1799A*, *TP53 c.G839C*, *TERT c.C228T* and *c.T349C*	Spindle; loosely associated spheroids
THJ-29T	*TP53 c.C310T* and *TERT c.C250T*	Spindle/giant; loosely associated spheroids

## Data Availability

The processed data are publicly available in a GitHub repository: https://github.com/darrentyson/ATC-3D-screen (accessed on 13 February 2022).

## Supplementary Materials

The following supporting information can be downloaded at: https://www.mdpi.com/article/10.3390/cancers14081855/s1, Figure S1: Spheroid images; Figure S2: Example of differential response in 2D vs. 3D cultures; Figure S3: Graphs of 2D vs. 3D activity area used to derive Figure 4C; Figure S4: Representative images of spheroids treated with 3 select compounds; Table S1: Summary of deprioritized compounds; Table S2: 2D vs. 3D activity area.
