# Supplementary material for: Utilizing Three-Dimensional Culture Methods to Improve High-Throughput Drug Screening in Anaplastic Thyroid Carcinoma
Source: Cancers (Basel). 2022 Apr 7;14(8):1855. doi: 10.3390/cancers14081855 (PMC9031362; doi:10.3390/cancers14081855)
Supplement: Supplementary file 1 [file cancers-14-01855-s001.zip › cancers-1617181-Suppl.Figure.pdf]

cancers MDPI

Supplementary Materials: Utilizing Three-Dimensional Culture Methods to Improve High-Throughput Drug Screening in Anaplastic Thyroid Carcinoma

Kensey Bergdorf, Joshua A. Bauer, David Westover, Courtney Phifer, Barbara Murphy, Darren R. Tyson, Ethan Lee and Vivian L. Weiss

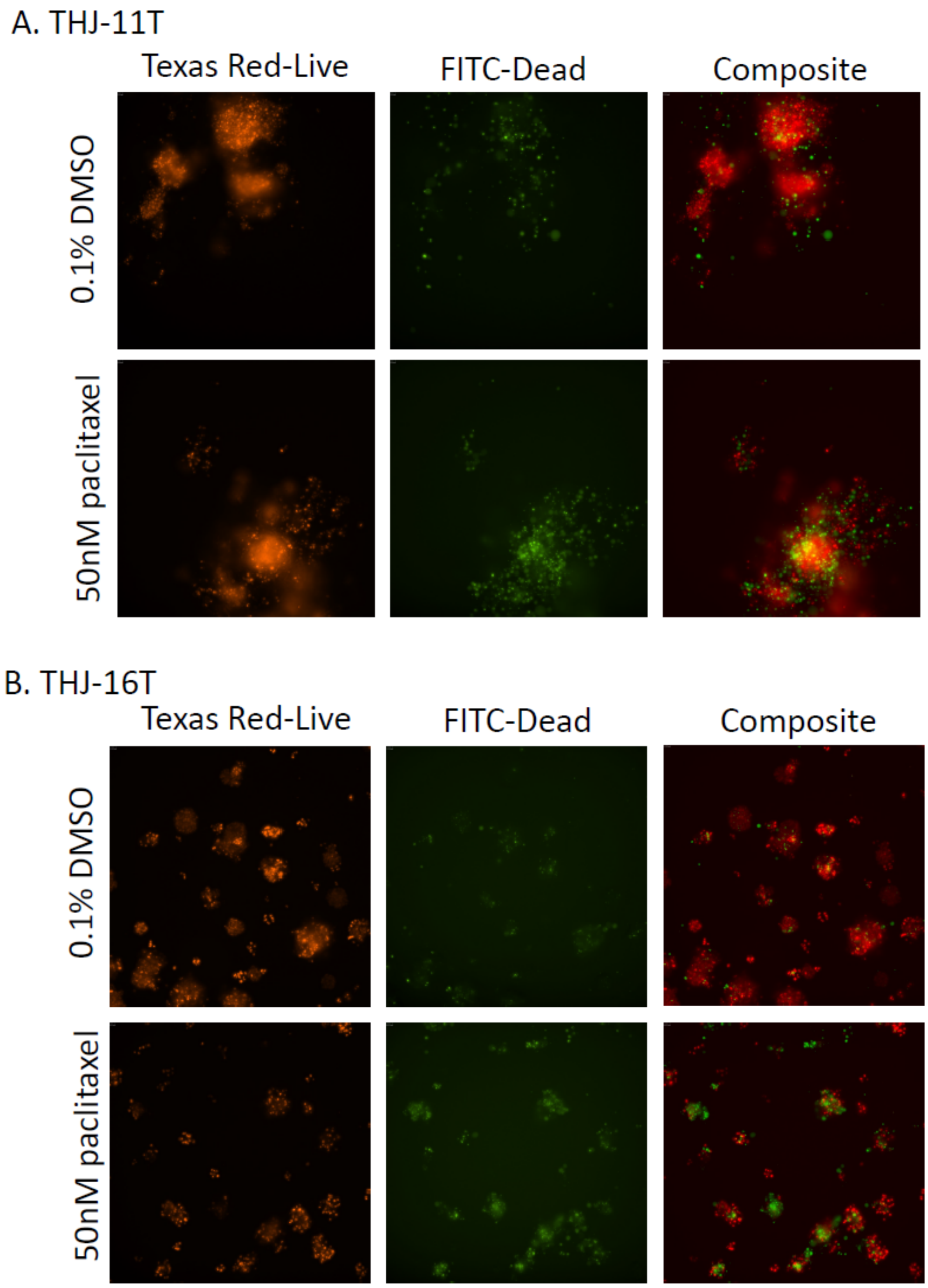

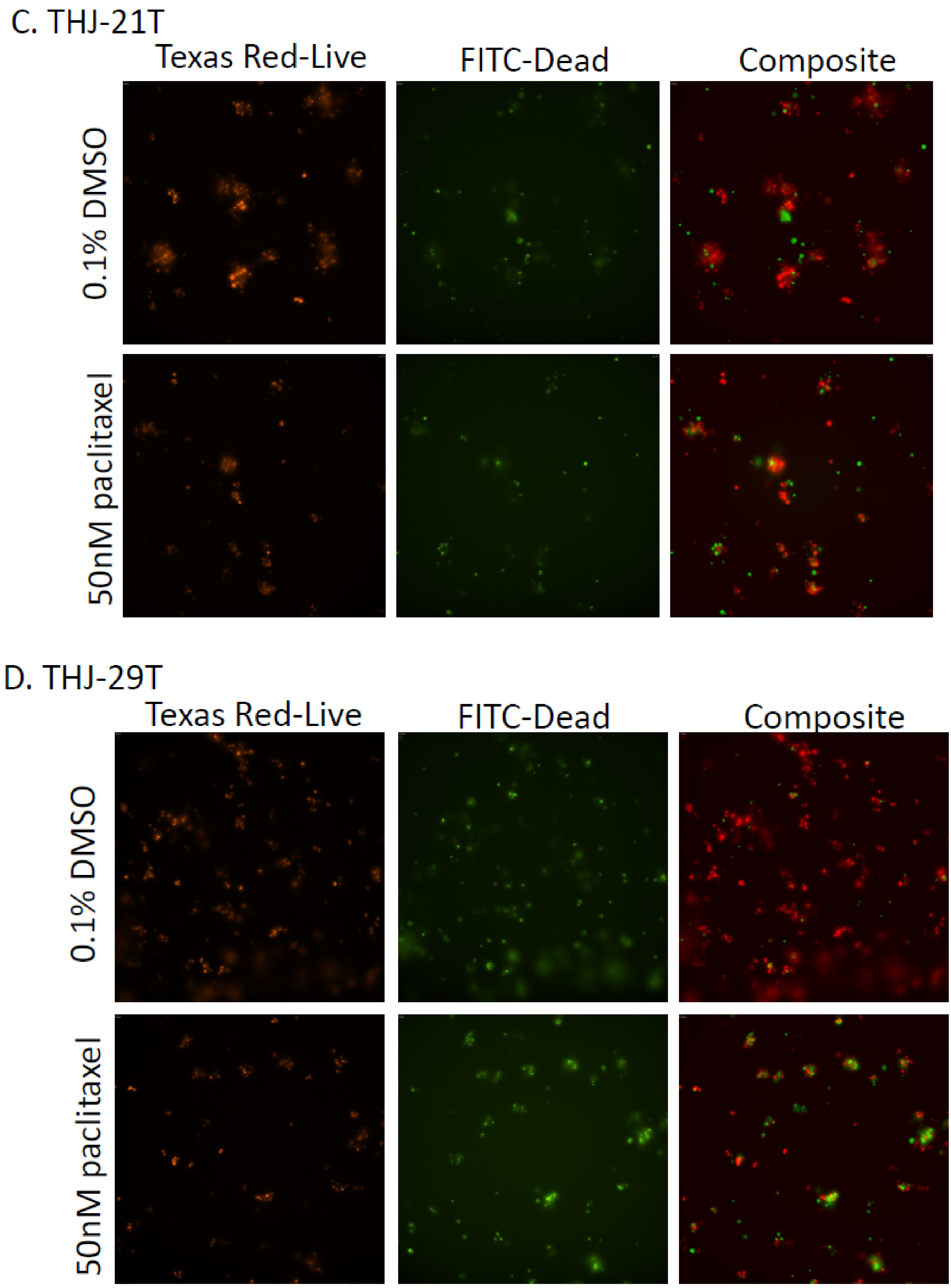

Figure S1. Spheroid images.

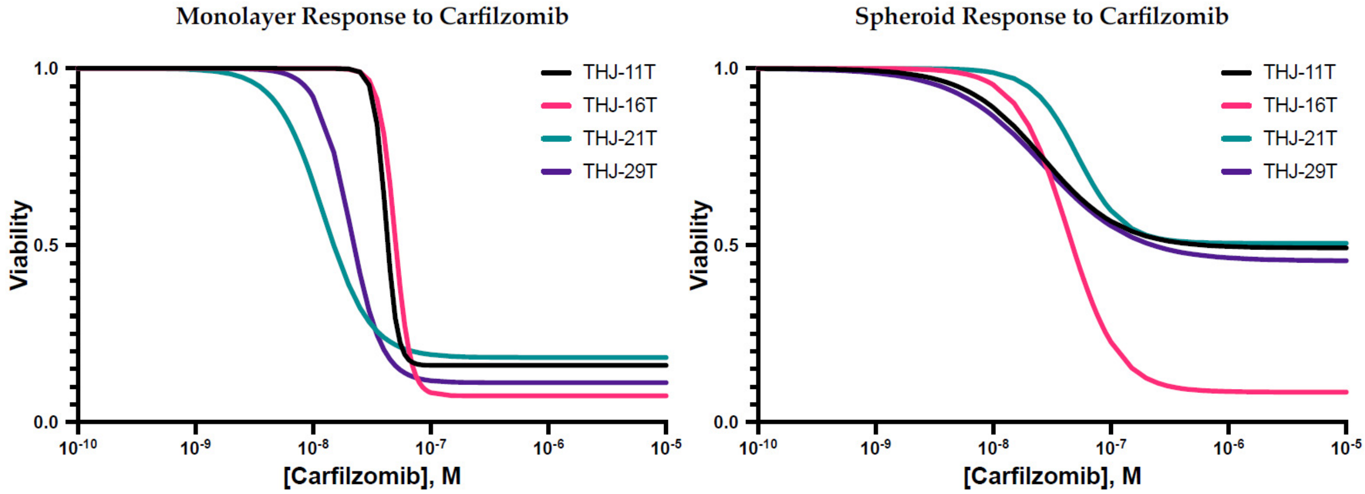

Figure S2. Example of differential response in 2D vs. 3D cultures.

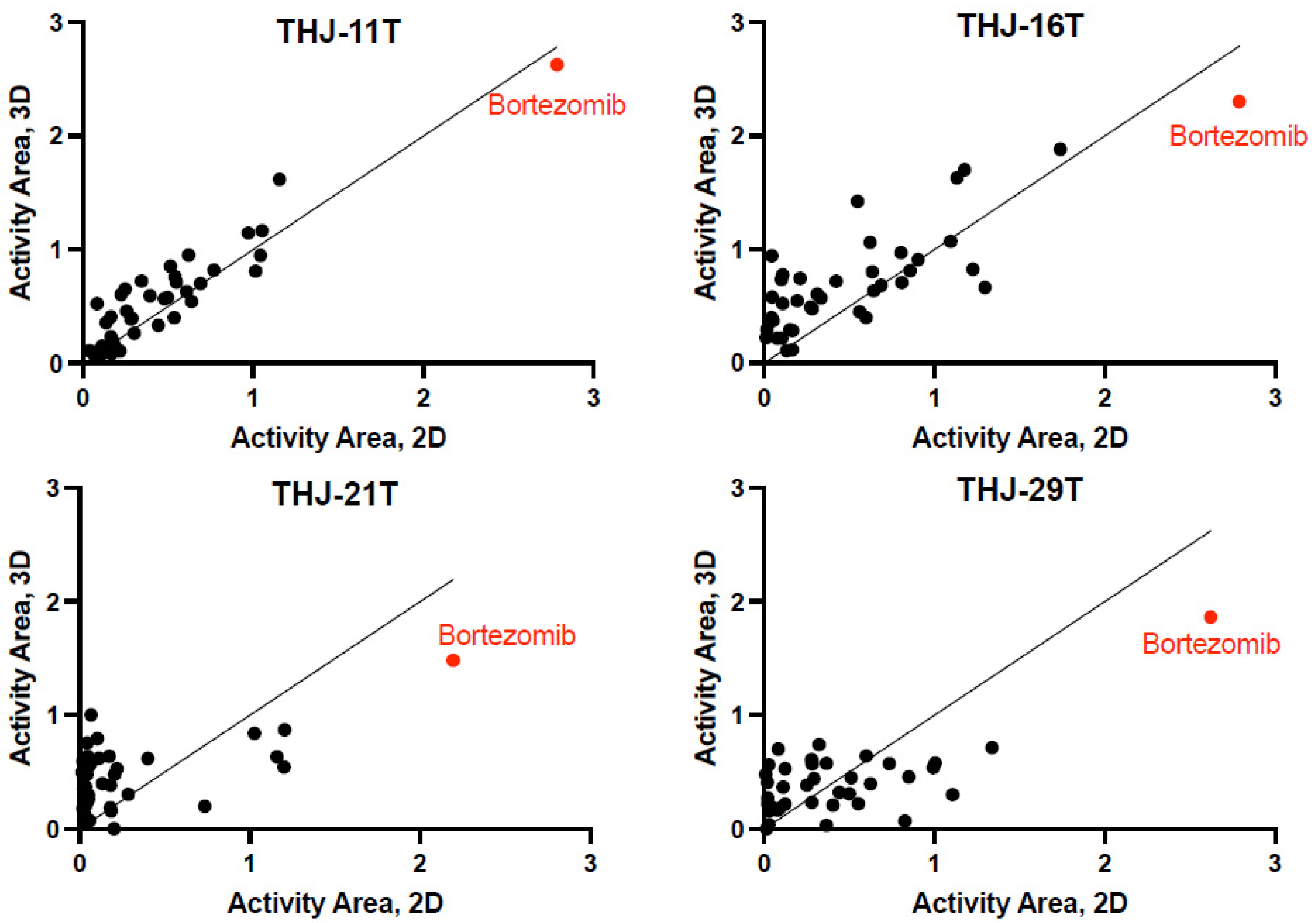

Figure S3. Graphs of 2D vs. 3D activity area used to derive Figure 4C.

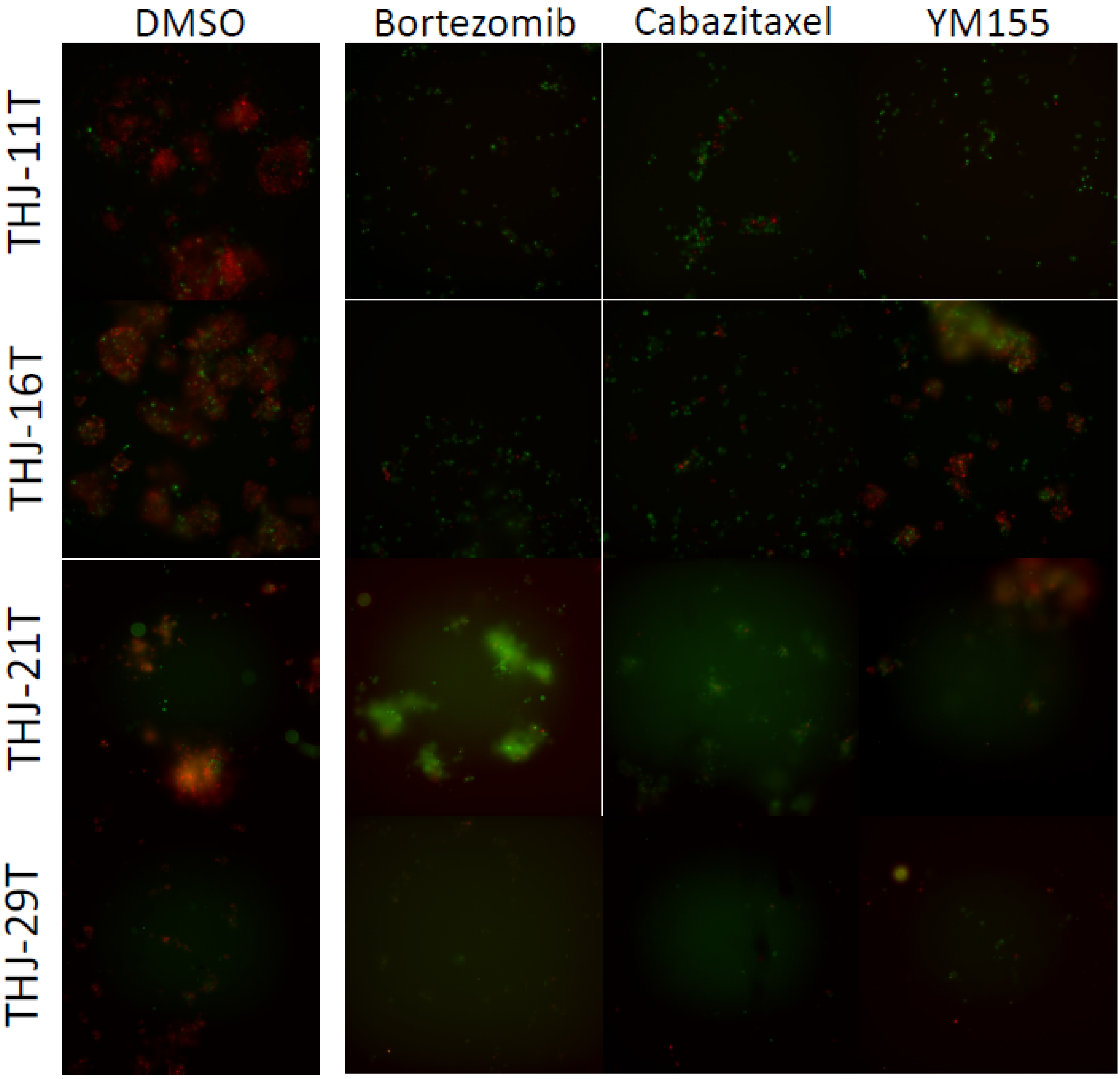

Figure S4. Representative images of spheroids treated with 3 select compounds.
